# Photobiomodulation Improves Serum Cytokine Response in Mild to Moderate COVID-19: The First Randomized, Double-Blind, Placebo Controlled, Pilot Study

**DOI:** 10.3389/fimmu.2022.929837

**Published:** 2022-07-08

**Authors:** Seyed Mehran Marashian, Mohammadreza Hashemian, Mihan Pourabdollah, Mansour Nasseri, Saeed Mahmoudian, Florian Reinhart, Alireza Eslaminejad

**Affiliations:** ^1^Chronic Respiratory Diseases Research Center, National Research Institute of Tuberculosis and Lung Diseases, Shahid Beheshti University of Medical Sciences, Tehran, Iran; ^2^Department of Immunology, School of Public Health, University of Medical Sciences, Tehran, Iran; ^3^National Research Institute of Tuberculosis and Lung Diseases (NRITLD), Shahid Beheshti University of Medical Sciences, Tehran, Iran; ^4^Medical Research & Innovation Department, Medical and Biomedical Consultancy Office “Innolys”, Illkirch-Graffenstaden, France

**Keywords:** covid-19, photobiomodulation, cytokine storm, IL-6, IL-8, IL-10, TNF-α, IL-6/IL-10 ratio

## Abstract

**Background:**

Because the major event in COVID-19 is the release of pre- and inflammatory cytokines, finding a reliable therapeutic strategy to inhibit this release, help patients manage organ damage and avoid ICU admission or severe disease progression is of paramount importance. Photobiomodulation (PBM), based on numerous studies, may help in this regard, and the present study sought to evaluate the effects of said technology on cytokine reduction.

**Methods:**

This study was conducted in the 2nd half of 2021. The current study included 52 mild-to-moderately ill COVID-19, hospitalized patients. They were divided in two groups: a Placebo group and a PBM group, treated with PBM (620-635 nm light *via* 8 LEDs that provide an energy density of 45.40 J/cm^2^ and a power density of 0.12 W/cm^2^), twice daily for three days, along with classical approved treatment. 28 patients were in Placebo group and 24 in PBM group. In both groups, blood samples were taken four times in three days and serum IL-6, IL-8, IL-10, and TNF-α levels were determined.

**Results:**

During the study period, in PBM group, there was a significant decrease in serum levels of IL-6 (-82.5% +/- 4, P<0.001), IL-8 (-54.4% ± 8, P<0.001), and TNF-α (-82.4% ± 8, P<0.001), although we did not detect a significant change in IL-10 during the study. The IL-6/IL-10 Ratio also improved in PBM group. The Placebo group showed no decrease or even an increase in these parameters. There were no reported complications or sequelae due to PBM therapy throughout the study.

**Conclusion:**

The major cytokines in COVID-19 pathophysiology, including IL-6, IL-8, and TNF-α, responded positively to PBM therapy and opened a new window for inhibiting and managing a cytokine storm within only 3-10 days.

## Introduction

COVID-19 is one of the most – if not the most – challenging pandemics in the modern world. It is caused by the SARS-CoV 2 coronavirus (1) and triggers severe acute respiratory syndrome (SARS). This condition is associated with overexpression of inflammatory markers such as interleukins (2).

Basically, there are two known phases of the immune response against COVID-19 in the body (3). The first phase belongs to B/T cell immunity and leads to the release of immunoglobulins (IgM and IgG) in the body. This is usually effective against a broad spectrum of infections, including bacterial, viral and fungal infections. The second phase, which is much more crucial in COVID-19 because it is an intracellular viral infection, results in the secretion of a variety of cytokines into the bloodstream by T cells and macrophages, of which IL1-β, IL-2, IL-8, IL-10, IL-6, IL-12, IFN-γ, and TNF-α are the best known (4, 5).

This second phase can be dramatically dysregulated in patients – sometimes referred to as a “cytokine storm” – leading to multiple organ failure, thromboembolism, and death (6). This is at least part of the explanation for the high mortality rate in COVID-19.

It is likely that the use of immunomodulatory agents could be a promising therapeutic strategy to reduce or even prevent this dysregulation and improve survival of moderate-to-severe patients by inhibiting or at least modulating the severe immune response to ARDS. Immunosuppressive drugs such as corticosteroids, a well-known group of immunomodulatory drugs, have been used along this pathway. However, their use may also increase the risk of secondary infections and prolongation of hospital stay (7, 8).

To date, there is no globally approved treatment for this “cytokine storm”. Therefore, there is an urgent need for new solutions. Here, we propose to test a non-drug approach to modulate the host immune response to improve the prognosis of patients.

According to previous studies, IL-6, IL-8, IL-10 and TNF-α seem to play an important role in the patient’s vital prognosis (9, 10). Therefore, in the present study, we focus on these 4. IL-6 is a pleiotropic cytokine that plays a crucial role in the pathophysiology of ARDS and leads to poor prognosis for patients (9). IL-8 is also involved in the pathophysiology of ARDS, neutrophil chemotaxis and lung tissue survival, and increases disease severity, resulting mortality in humans (10). IL-10 is mainly known as an anti-inflammatory cytokine, especially in chronic diseases such as (not conclusive) chronic obstructive pulmonary disease (COPD) (11), tuberculosis (12), rheumatoid arthritis (13), etc. IL-10 may actually modulate the production of inflammatory cytokines to reduce the risk of tissue damage (14–16). TNF-α is an important proinflammatory marker involved in several immune pathways, such as neutrophil adhesion and activation, but also in the production of IL-6 and in the development of edema due to coagulation in acute pneumonia (17, 18).

Photobiomodulation (PBM), also known as low-level light therapy (LLLT), has been studied and used for over 50 years (19–26) to protect cells from dying (22, 24, 27–30) and to target and treat inflammation in organs (31, 32). It consists of the application of a narrow spectral bandwidth light from red to near-infrared light (600-1000 nm wavelength) with a power density of 1-5000 mW/cm^2^ to modulate cell metabolism, signal transduction, and secretion in the body (33–35).

Since PBM has shown promising effects on inflammation control in many medical situations (15, 31, 36–40), such as ARDS in the lung (10), it seems to be an alternative approach of choice to address the need for immune regulation in moderate to severe COVID-19 patients (41).

De Lima et al. indicated that PBM effectively affects TNF-α reduction in alveolar macrophages to reduce the incidence of ARDS in mice (35). PBM has also been shown to inhibit the migration of neutrophils into lung tissue, resulting in less activation and higher apoptosis of these cells to reduce neutrophil accumulation and ultimately alleviate the severity of pneumonia and ARDS (10). Some studies have shown that PBM leads to a decrease in IL-6 in serum and lung tissue in COPD patients, while increasing the CD4^+^/CD8^+^ ratio (10, 42, 43), and decreasing the risk of fibrosis in chronic pneumonia (36).

Based on the above findings, the current study sought to investigate the clinical effects of PBM on mild and moderate COVID-19 cases hospitalized in a tertiary college referral center for respiratory and infectious diseases in Tehran.

## Materials and Methods

### Subjects

A total of 52 patients with COVID-19 participated in this double-blind, randomized, placebo-controlled study from Masih Daneshvari Hospital in Tehran, Iran. The World Health Organization (WHO) global guideline was used to select cases (44). All patients suffered from COVID-19 (due to a positive PCR test) and required hospitalization. They complained of various symptoms such as fever, malaise, chills, fatigue, and some showed additional respiratory symptoms and signs such as dyspnea, cough, and arterial O_2_ saturation of no more than 90% and pulmonary involvement of less than 50%. We excluded individuals with ARDS, chest pain, and respiratory rate ≥ 30 breaths per minute and accessory respiratory muscle actuation. We also excluded chronic immune diseases or medical situations affecting the immune system or individuals taking immunosuppressive drugs such as corticosteroids, tocilizumab or other similar drugs. None of the participants were mechanically ventilated in one way or another, although almost all required oxygen *via* nasal tubes or face masks.

### Ethics

The current study was approved by the Iranian Registry of Clinical Trials under reference number IRCT20200616047799N1. The investigators explained the aim and procedure of the current study to each patient before enrollment. Each participating patient signed an informed consent form. There was no restriction on participants who wished to withdraw from the study. All participants took their COVID-19 treatments according to the protocol, and individuals who required corticosteroids and/or effective immune system medications such as tocilizumab (Actemra) were excluded to avoid having to give up these medications. If side effects of PBM therapy were observed, patients were expected to be excluded from the study; however, this was not the case. No additional costs or unnecessary procedures were imposed on participants, and all times of blood collection were adjusted to fit the care routine as much as possible.

### Randomization and Blinding Processes

Before the start of the study, subjects were randomly divided into two groups: (1) placebo control group (Placebo) and (2) PBM treated group (PBM). Subjects in all groups were enrolled at Masih Daneshvari Hospital. To ensure blinding of the study, a physician from the clinical department was responsible for the blinding process. He had no contact with the research team at any time during the entire study. He was the only person who operated the devices. The researchers responsible for the care of the patients also had no knowledge of the assignment of the subjects to the groups, thus ensuring impartiality in the evaluations. Patients were blinded with dark glasses and noise-canceling headphones during the few minutes that the PBM devices were in operation.

### PBM and Placebo Treatment Protocols

**Figure 1:**
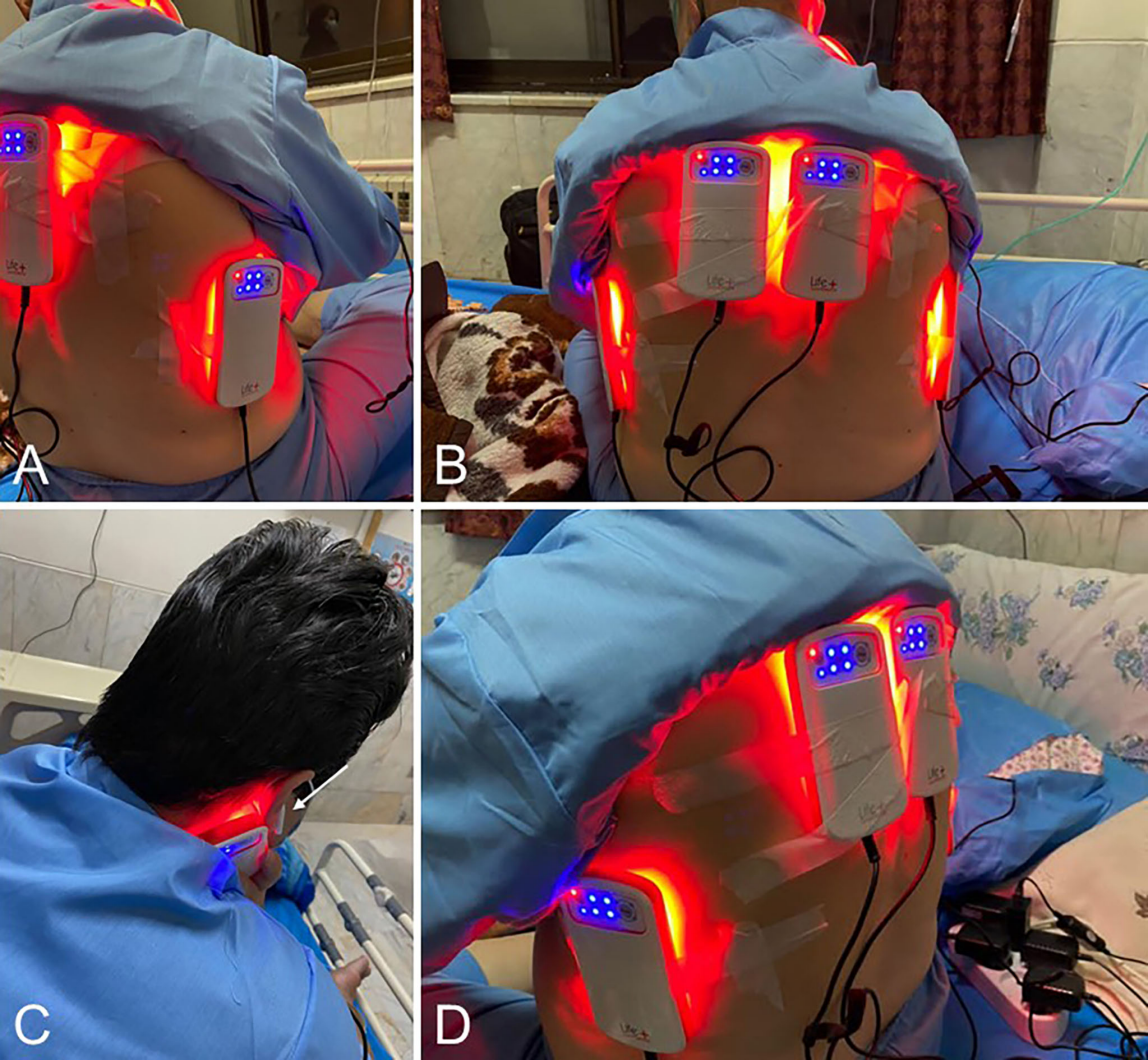
Illustrations of the positioning of the Photobiomodulation devices on the patients. **(A, D)**, 3/4 back view. **(B)**, Back view. **(C)**, View of the device positioned at the carotid artery; the white arrow shows the patient’s sound isolation device for blinding.

**Figure 2:**
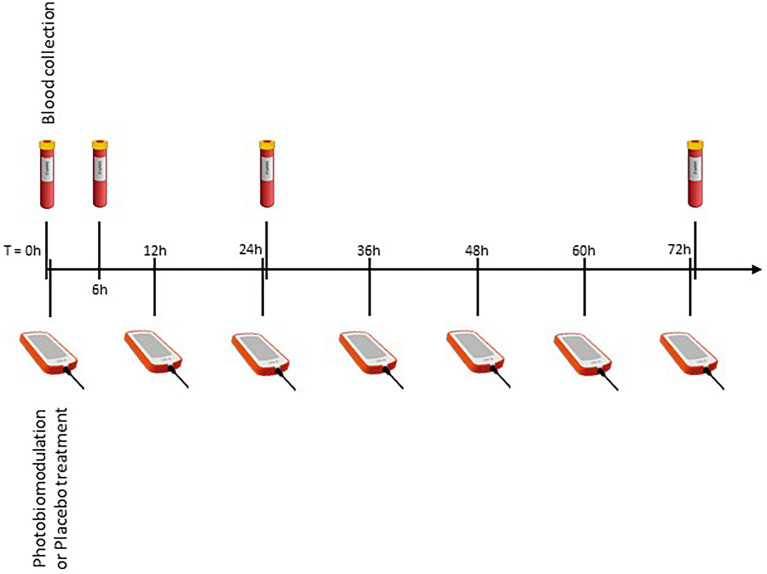
Overview of the experimental protocol. Four blood samples, at different time points, to measure inflammatory markers, and seven illuminations (every 12 hours).

The “LifePlus^®^ NeolysPlus^®^” devices were provided by Elorabiotec^®^ and developed/produced by Xbiotec^®^ using Actycel^®^ technology. As shown in **Figure 1**, the devices were deployed at five sites on the body, including both sides of the thoracolumbar regions, both sides of the anterolateral chest wall, and one site on the neck at the carotid arteries. The devices emitted a red light with a wavelength of 620-635 nm *via* 8 LEDs, providing an energy density of 45.40 J/cm^2^ and a power density of 0.12 W/cm^2^ when irradiated. Five devices were attached to participants’ bodies at the specified locations and turned on for 6 minutes for the PBM group. The procedure was repeated every 12 hours for three consecutive days (see **Figure 2**). The same procedure was used for the Placebo group, but without turning on the device, only to mimic the handling. Treatment with PBM or placebo began as soon as patients were admitted to the hospital.

To prevent patient-to-patient transmission of infection, we used disposable transparent sheets in which we placed the devices before placing them on the skin. The devices turned off automatically after exactly 6 minutes, and no one absorbed less or more radiation.

### Blood Sampling and Laboratory Analysis

Routinely, blood samples are collected every day at 6:00 am for all patients in the wards COVID of the hospital. We created a table for sample collection, taking into account ward discipline to have the fewest samples for patients. Blood samples were needed immediately before the procedure (zero time) and 6, 24, and 72 hours after zero time (T0, T6, T24, T72, respectively, see **Figure 2**). Whole blood was collected by venipuncture into 5mL clot activation tubes. After clot activation, the serum tubes were spun in a refrigerated centrifuge at 3000 rpm for 10 minutes. After centrifugation, the serum was aseptically filled into cryotubes and frozen at -70°C until assayed.

Serum cytokines, including IL-6, IL-8, IL-10 and TNF-α, were measured in an advanced immunology and genetics laboratory using “ELISA kits, by R&D^®^, USA”, namely DY206-05, DY208-05, DY217B-05 and DY210-05.

### Outcome Measure

The described percentage deviations in the results section were obtained by normalizing the raw data to the measurements before the protocol (T0).

Measurement of IL-6 level is the primary end point of this study, whereas IL-8, IL-10, and TNF-alpha are the secondary end points. The current study sought to evaluate the efficacy of PBM in ameliorating or completely eliminating cytokine dysregulation in patients.

Considering the short duration of the study (3 days), a reduction in serum levels of IL-6 by ≥ 40% and other levels by ≥ 20% would be considered a positive outcome.

Importantly, the current study focused on some cytokine profiles that were affected by PBM (which is not an antiviral technique). Viral load was not a confounding factor for us in this regard. We tried to measure serum cytokine levels rather than viral load. As far as we know, there is no documentation of an absolute and strict correlation between viral load and cytokine levels, at least not in COVID-19.

### Statistics

The present study used a randomized clinical design with PBM and Placebo groups. All quantitative variables were expressed as mean ± standard deviation, whereas qualitative variables were presented in numbers (percent). For comparison of quantitative variables, the Student t-test was used, and the Mann-Whitney test was to be used as a nonparametric test when necessary (when the normality hypothesis was not met – this case was not observed).

For qualitative variables, the Pearson chi-square test and Fisher’s exact test were used. For longitudinal data, Repeated Measure ANOVA was used, with covariates defined as variable values at baseline to adjust for the effects of the last item. SPSS 26 was used for the entire analysis with a 95% confidence interval and a significance level of 0.05.

## Results

A total of 52 participants were enrolled in the study, 28 in the Placebo group and 24 as PBM (see **Table 1**). The mean age was 54.5 ± 13.56 years in the Placebo group and 53.88 ± 18.28 years in the PBM group, with no significant difference between the groups (P=0.578). There were 11 (45.8%) men and 13 (54.2%) women in the PBM group, whereas the sex ratio in the Placebo group was 15 (53.6%) men and 13 (46.4%) women. There was no significant difference between the two groups (P=0.888).

**Table 1:** Sex and age distribution between the two groups.

	Placebo	PBM	P-Value between groups
**Sex**			0.578
**Male**	15 (53.6%)	11 (45.8%)	
**Female**	13 (46.4%)	13 (54.2%)	
**Age**	54.50 ± 13.56	53.88 ± 18.28	0.888

Repeated ANOVA showed no effect of age and sex on biomarkers measured in the current study: IL-6 (age, P=0.984; sex, P=0.775), IL-8 (age, P=0.894; sex, P=0.423), IL-10 (age, P=0.927; sex, P=0.734), TNF-α (age, P=0.423; sex, P=0.667).

Raw data for IL-6, IL-8, IL-10 and TNF-α levels are summarized in **Table 2**. Briefly, statistical analysis showed no difference between groups at T0 for IL-6 (P=0.776), IL-8 (P=0.963), IL-10 (P=0.051), and TNF-α (P=0.557). At T72, the PBM group showed lower values for IL-6 (P=0.001) and IL-8 (P=0.003). TNF-α is significantly lower in the PBM group at T24 (P=0.044) and T72 (P=0.008). However, there is no difference between groups in terms of IL-10 values at T72 (P=0.468).

**Table 2:** Raw values (mean +/- SEM) for IL-6, IL-8, IL-10, and TNF-α secretion levels at T=0, 6, 24, and 72 h, of PBM and Placebo groups.

	Placebo	PBM	P-Value between groups
**IL-6**			
**0 hours**	41.91 ± 24.10	99.05 ± 43.45	0.776
**6 hours**	51.21 ± 23.02	49.95 ± 28.82	0.393
**24 hours**	52.11 ± 24.61	25.31 ± 12.38	0.036
**72 hours**	45.06 ± 23.38	17.38 ± 8.16	0.001*
**IL-8**			
**0 hours**	16.20± 1.90	26.14 ± 8.37	0.963
**6 hours**	18.62 ± 1.99	18.57 ± 4.34	0.202
**24 hours**	18.54 ± 2.19	16.65 ± 3.78	0.086
**72 hours**	20.83 ± 2.97	11.93 ± 2.93	0.003*
**IL-10**			
**0 hours**	46.05 ± 29.60	123.53 ± 44.36	0.051
**6 hours**	50.55 ± 35.16	83.29 ± 31.20	0.209
**24 hours**	44.00 ± 28.65	91.78 ± 37.21	0.226
**72 hours**	36.24 ± 21.84	95.19 ± 38.15	0.468
**TNF-α**			
**0 hours**	29.94 ± 16.10	64.33 ± 47.56	0.557
**6 hours**	37.35 ± 23.18	16.37 ± 6.31	0.430
**24 hours**	29.63 ± 14.88	14.16 ± 8.00	0.044*
**72 hours**	30.95 ± 17.58	11.31 ± 6.01	0.008*

*means the difference between groups is statistically significant (P<0.05).

### Effect of Photobiomodulation on IL-6 Secretions

**Figure 3:**
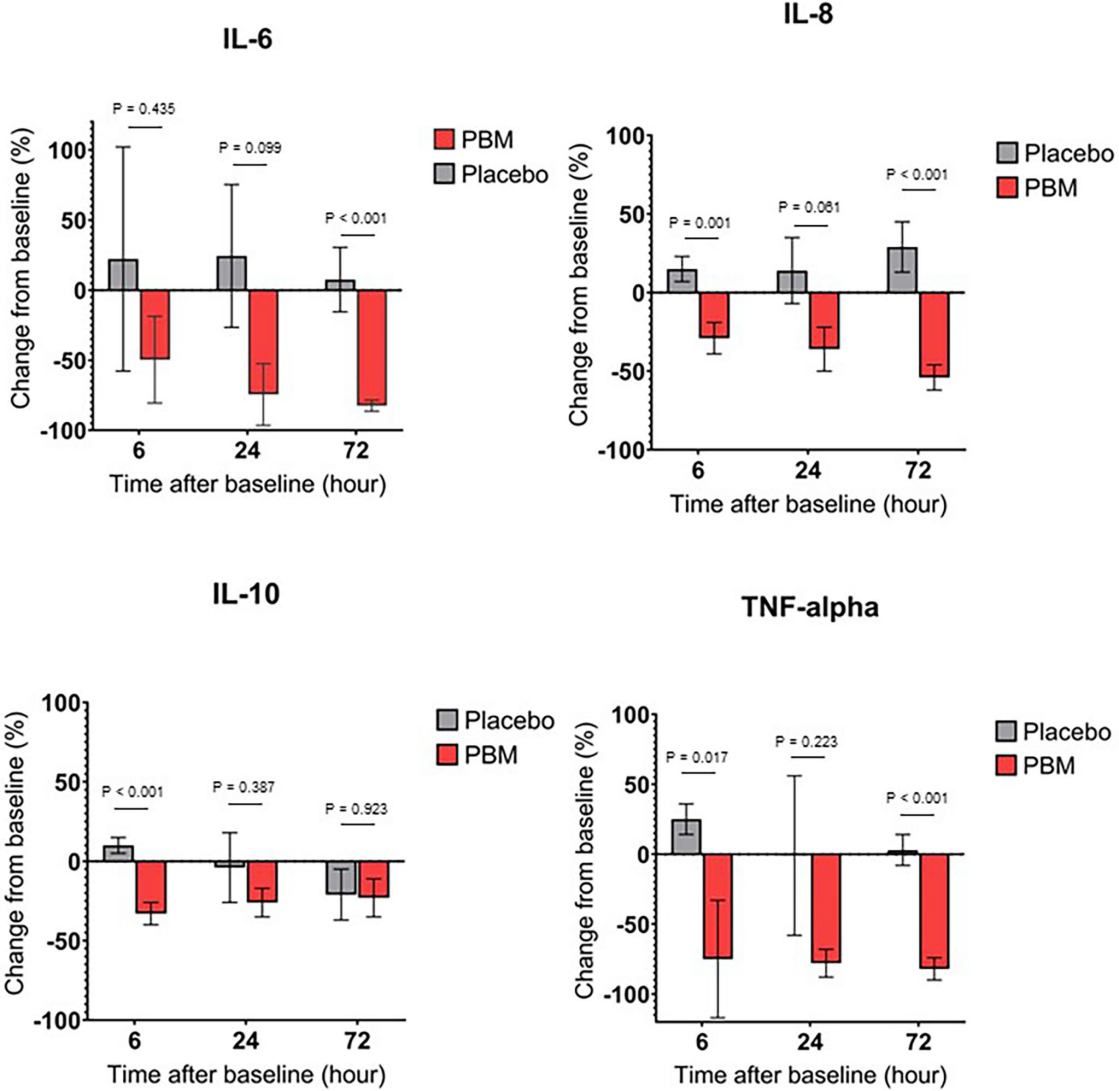
Cytokine secretion changes over time, for Placebo and PBM groups compared to T0 (Baseline). (A) IL-6. (B) IL-8. (C) IL-10. (D) TNF-alpha.

As shown in **Figure 3A** and **Table 3**, IL-6 shows a tendency to increase in the Placebo group at T6 and T24 hours but not significantly (respectively, +22.2% ± 80, P=0.782; and +24.3% ± 51, P=0.635). In the PBM group, there is a trend toward a decrease at T6, but this is not significant (-49.6% ± 31, P=0.117). This decrease reaches significance at T24 (-74.5% ± 22, P=0.001). Moreover, there is no difference between the two groups, neither at T6 (P=0.435) nor at T24 (P=0.099).

**Table 3:** Percentage of change in secretion levels of IL-6, IL-8, IL-10 and TNF-alpha, after 6, 24 and 72h of PBM or Placebo (based on normalized values to T=0h).

	Placebo	PBM	P-Value between groups
**IL-6**			
**6 hours**	+22.2% ± 80	-49.6% ± 31	0.435
**24 hours**	+24.3% ± 51	-74.5+ ± 22	0.099
**72 hours**	+7.5% ± 23	-82.5% ± 4	<0.001*
**IL-8**			
**6 hours**	+14.9% ± 8	-29.0% ± 10	0.001*
**24 hours**	+14.4% ± 21	-36.3% ± 14	0.061
**72 hours**	+28.5% ± 16	-54.4% ± 8	<0.001*
**IL-10**			
**6 hours**	+9.8% ± 5	-32.6% ± 7	<0.001*
**24 hours**	-4.5% ± 22	-25.7% ± 9	0.387
**72 hours**	-21.3% ± 16	-22.9% ± 12	0.923
**TNF-α**			
**6 hours**	+24.7% ± 11	-74.6% ± 42	0.017*
**24 hours**	-1.0% ± 57	-78.0% ± 10	0.223
**72 hours**	+3.4% ± 11	-82.4% ± 8	<0.001*

A positive value represents an increase in secretion levels, while a negative value represents a decrease. * means the difference between groups is statistically significant (P<0.05).

At T72, the end of the study, the IL-6 value has not changed significantly in the Placebo group (+7.5% ± 23, P=0.745), whereas it has decreased significantly in the PBM group (-82.5% ± 4, P<0.001).

Overall, IL-6 did not change in the Placebo group and decreased in the PBM group over the course of the study.

### Effect of Photobiomodulation on IL-8 Secretions

IL-8 showed a tendency to increase, thus not significantly other the course of the study in the Placebo group (T6: +14.9% ± 8, P=0.066; T24: +14.4% ± 21, P=0.508; T72: +28.5% ± 16, P=0.075). On the other hand, in the PBM group, IL-8 is significantly decreased at T6 (-29.0% ± 10, P=0.006), T24 (-36.3% ± 14, P=0.013), and T72 (-54.4% ± 8, P<0.001).

In addition, the PBM group is significantly lower than the Placebo group at T6 (P=0.001) and T72 (P<0.001).

Overall, IL-8 did not change in the Placebo group and decreased in the PBM group over the course of the study. All these results are summarized in **Figure 3B** and **Table 3**.

### Effect of Photobiomodulation on IL-10 Secretions

As shown in **Figure 3C** and **Table 3**, IL-10 in the placebo group shows no difference from baseline at T6 (+9.8% ± 5, P=0.051), T24 (-4.5% ± 22, P=0.856), or T72 (-21.3% ± 16, P=0.195). The PBM group shows a significant decrease at T6 (-32.6% ± 7, P<0.001) and at T24 (-25.7% ± 9, P=0.006) but no difference from baseline at T72 (-22.9% ± 12, P=0.062).

The PBM group is significantly lower than Placebo at T6 (P<0.001), but there is no difference between the two groups at either T24 (P=0.387) or T72 (P=0.923).

### Effect of Photobiomodulation on TNF-α Secretions

The Placebo group showed a significant increase in TNF-α levels from baseline (**Figure 3D**, **Table 3**) at T6 (+24.7% ± 11, P=0.027). There was no difference from baseline at either T24 (-1.0% ± 57, P=0.986) or T72 (+3.4% ± 11, P=0.786). The PBM group showed a tendency to decrease, but not significantly at T6 (-74.6% ± 42, P=0.081), and reached significance at T24 (-78.0% ± 10, P<0.001) and T72 (-82.4% ± 8, P<0.001).

TNF-α levels are significantly lower in the PBM group than in the Placebo group at T6 (P=0.017) and T72 (P<0.001).

### Effect of Photobiomodulation on IL-6/IL-10 Ratio

**Figure 4:**
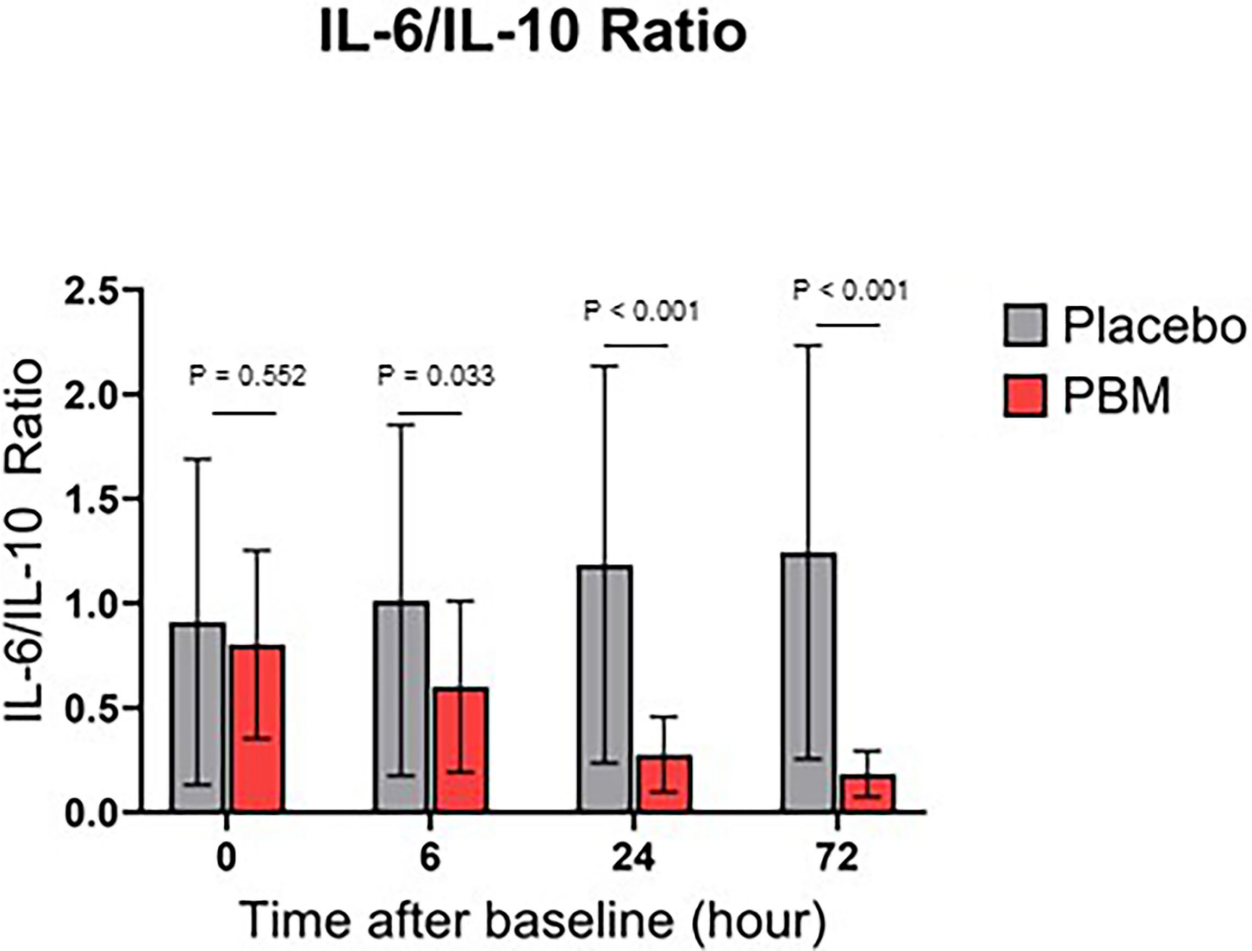
IL-6/IL-10 Ratio over time, for Placebo and PBM groups.

IL-6/IL-10 ratio for both groups are shown in **Figure 4**. Briefly, the Placebo group showed no significant change in the ratio IL-6/IL-10, neither at T6 (P=0.636), nor at T24 (0.243), nor at T72 (P=0.167). On the other hand, PBM showed a significant improvement in its IL-6/IL-10 ratio at T24 (P<0.001) and T72 (P<0.001).

In addition, the IL-6/IL-10 ratio is significantly lower in the PBM group than in the Placebo group at every time point of the study, except at baseline (T0: P=0.552; T6: P=0.033; T24: P<0.001; T72: P<0.001).

## Discussion

The present study sought to evaluate the effects of PBM on selected serum biomarkers in a cohort of COVID-19 patients with mild to moderate disease who did not require mechanical ventilation or intensive care.

To our knowledge, the present study is the first randomized, double-blind, placebo-controlled trial to demonstrate the therapeutic potential of PBM on the inflammatory phase of COVID-19. Indeed, our results show a significant decrease in the levels of IL-6, IL-8, and TNF-α in PBM patients compared with their levels at enrollment and compared with Placebo patients after 72 hours of treatment. PBM patients also show an improvement in the IL-6/IL-10 ratio compared with Placebo patients, as early as 6 hours after the start of treatment.

Our findings are consistent with observations made by Sigman et al. in their case study of a 57-year-old African-American man in 2020 with a history of hypertension, asthma, and acute renal failure who was hospitalized for severe COVID-19 with SpO_2 _= 80%. They suggested that the use of PBM therapy early in the disease improves recovery, prevents lung tissue damage and ICU admission, while significantly reducing recovery time and oxygen requirements (45).

Our results also complement numerous preclinical and clinical studies in which the anti-inflammatory potential of PBM has been observed. For example, Brochetti et al. showed that PBM improved inflammatory and fibrotic parameters in a mouse model of pulmonary fibrosis (31). The results were recently confirmed by Brito et al. (36) in a model of idiopathic fibrosis: this team showed that PBM reduced the secretion of TNF-α, IL-1β, IL-6 and interferon gamma (IFNγ). Similar results were shown in models of acute lung injury after sepsis (decreased TNF-α, IL-1β, and IL-6 secretion) (38, 39) and paraformaldehyde injury (decreased cell death, MyeloPeroxidase activity, parenchymal vascular permeability, TNF-α and IL-6 secretion, and increased IL-10 secretion) (15, 40).

In humans, several studies have reported the importance of PBM as an anti-inflammatory treatment, stabilizing immune response, improving mechanical ventilation, reducing clinical symptoms and length of hospital stay in chronic lung diseases such as bronchial asthma (46), and chronic obstructive pulmonary disease (COPD) (47). Specifically, in a double-blind, randomized, placebo-controlled, crossover clinical trial, de Souza et al. showed that PBM improved exercise capacity in COPD patients (11).

Our results in humans COVID+ are very similar, especially with respect to TNF-α, IL-8 and IL-6. Measurement of IL-6 was our main evaluation criterion because its changes in the COVID-19 (48) situation are prognostic and require close monitoring and control to achieve optimal treatment response and outcome. The ability of PBM to decrease IL-6 levels in patients is therefore strong evidence of the anti-inflammatory potential of this strategy in the fight against severe forms of COVID-19, and its importance in the therapeutic armamentarium.

A notable difference between the previously described studies and our results is the lack of change in IL-10 values in the PBM group compared with the Placebo groups. Although not significant (except for T6), our results suggest a trend toward lower IL-10 levels in PBM patients. IL-10 is primarily known as an anti-inflammatory cytokine: in the face of infection, IL-10 primarily inhibits the host immune response to pathogens. Consequently, it attenuates tissue damage and immunopathology by inhibiting the synthesis of proinflammatory cytokines (such as IFNγ, IL-2, IL-3, TNFα, and G-CSF31) (37, 49, 50).

Thus, at first glance, the tendency for PBM to decrease IL-10 in our study does not seem to confirm these studies. However, several studies suggest that the level of IL-10 in COVID-19 should not be considered alone, but in conjunction with the secretion of other cytokines, especially IL-6 (51). Zeng et al. (52) suggest that the increase in IL-10, although a marker of the anti-inflammatory response to the abrupt increase in proinflammatory cytokines, may be a sign of worsening disease. Indeed, the authors suggest that a persistently elevated IL-10 correlates with a poor prognosis for the patient. Similar observations have been made in patients with sepsis, where overproduction of IL-10 appears to be a predictor of disease severity and adverse outcome (53, 54). IL-10 may also cause immunosuppression when overexpressed (55). Together with these studies, our results suggest that PBM may prevent overexpression of IL-10 and thus promote a favorable prognosis for patients. However, further studies are needed to confirm the trend we observed.

In addition, comparison of the IL-6/IL-10 ratio of Placebo and PBM patients shows a significant improvement in PBM patients compared with baseline and to Placebo patients. This suggests the ability of PBM to regulate the cytokine network. Indeed, several studies show an association between the reduction of this ratio and the improvement of patients’ prognosis (56–58). Moreover, Azaiz et al. (51) classified patients according to their outcome (death, severe cases, mild cases, etc.) and their IL-6 and IL-10 scores. Their results show a higher incidence of severe and even fatal forms of COVID-19 when IL-6 and IL-10 are high. Conversely, patients with low IL-6 and IL-10 values are more likely to have mild forms of the disease without the need for mechanical ventilation. Taken together, these data support the idea of a therapeutic anti-inflammatory potential of PBM observed in our study. Indeed, our results suggest an anti-inflammatory and cytokine secretion regulating effect of PBM.

One of the limitations of the current study is its short duration. We aimed to perform this pilot study on the therapeutic potential of PBM on the inflammatory parameters of COVID-19 in patients with mild to moderate severity. Due to the outbreak of an epidemic in the catchment area of our hospital at the time of the study, we could not keep these patients in the hospital for more than three days. Indeed, their clinical picture did not allow for a longer hospital stay. We therefore opted for a short-term study in order not to monopolize beds that would otherwise be needed for more severely affected patients. A study of longer duration and with a larger number of patients would allow us to establish a clear link between the ability of PBM to regulate the cytokine network and the prognosis of patients, which cannot be answered in our study.

In conclusion, the major cytokines in COVID-19 pathophysiology, including IL-6, IL-8, and TNF-α, are significantly improved by Photobiomodulation, as is the IL-6/IL-10 ratio. Further studies with longer duration, larger number of patients and in patients with more severe forms of the disease are now needed to confirm our promising results.

## Data Availability Statement

The original contributions presented in the study are included in the article/supplementary material. Further inquiries can be directed to the corresponding author.

## Ethics Statement

The studies involving human participants were reviewed and approved by ethics committee of Shahid Beheshti University of Medical Sciences. The patients/participants provided their written informed consent to participate in this study.

## Author Contributions

SM: methodology, study design, research supervision, and report affairs; MH: case selection; MP: laboratory supervisor; MN: immunologic laboratory affairs; SM: laboratory acts; FR: scientific supervisor, study design, manuscript proofreading, and data analysis; AE: clinical supervisor and case selection. All authors contributed to the article and approved the submitted version.

## Funding

The salaries and contributions of the different authors were covered by their respective employers. Photobiomodulation devices have been kindly provided by Elorabiotec, just for the study duration. Elorabiotec also paid the Elisa kit.

## Conflict of Interest

Author FR is employed by Innolys. The remaining authors declare that the research was conducted in the absence of any commercial or financial relationships that could be construed as a potential conflict of interest.

## Publisher’s Note

All claims expressed in this article are solely those of the authors and do not necessarily represent those of their affiliated organizations, or those of the publisher, the editors and the reviewers. Any product that may be evaluated in this article, or claim that may be made by its manufacturer, is not guaranteed or endorsed by the publisher.
